# Fabrication of Coaxial and Confocal Transducer Based on Sol-Gel Composite Material for Optical Resolution Photoacoustic Microscopy

**DOI:** 10.3390/diagnostics10010006

**Published:** 2019-12-21

**Authors:** Masayuki Tanabe, Tai Chieh Wu, Makiko Kobayashi, Che Hua Yang

**Affiliations:** 1 Faculty of Advanced Science and Technology, Kumamoto University, Kumamoto 8608555, Japan; 2 College of Mechanical and Electrical Engineering, National Taipei University of Technology, Taipei 10608, Taiwan

**Keywords:** ultrasonic transducer, photoacoustic imaging, optical-resolution photoacoustic microscopy, sol-gel composite material

## Abstract

We have newly developed coaxial and confocal optical-resolution photoacoustic microscopy based on sol-gel composite materials. This transducer contains a concave-shaped piezoelectric layer with a focus depth of 5 mm and a hole with a diameter of 3 mm at the center to pass a laser beam into a phantom. Therefore, this system can directly detect an excited photoacoustic signal without prisms or acoustic lenses. We demonstrate the capability of the system through pulse-echo and photoacoustic imaging experiments. The center frequency of the fabricated transducer is approximately 7 MHz, and its relative bandwidth is 86%. An ex-vivo experiment is conducted, and photoacoustic signals are clearly obtained. As a result, 2- and 3-dimensional maximum amplitude projection images are reconstructed.

## 1. Introduction

Photoacoustic (PA) imaging is a novel visualization method that provides optical resolution and acoustic penetration. A PA imaging system employs a pulsed laser to generate acoustic waves at a focal point and an ultrasonic transducer to detect emitted acoustic waves [1,2,3,4,5,6,7]. Optical resolution PA microscopy (OR-PAM) is a PA imaging method where the optical focal size is considerably smaller than that of ultrasound, and thus, the spatial resolution of OR-PAM is generally determined by optical diffraction. The signal-to-noise ratio (SNR) and sensitivity of PA signals mainly depend on energy power required to transmit a pulsed laser and the receiving performance of an optic-acoustic transmitter (OAT), which transmits a pulsed laser and receives PA signals.

**Figure 1:**
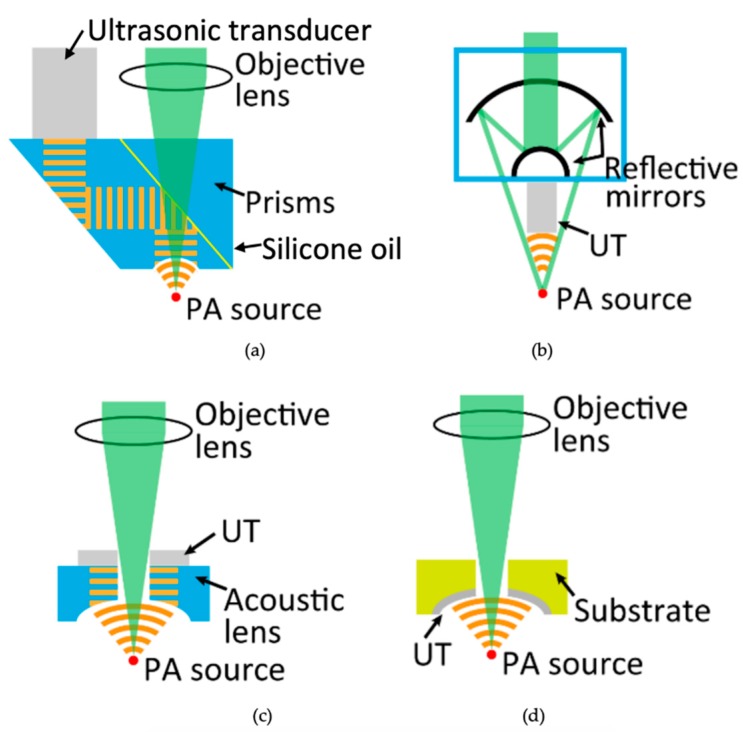
Optical-acoustic combinations in optical-resolution photoacoustic microscopy (OR-PAM). (**a**) Piston ultrasonic transducer (UT) is used with a two-prism acoustic reflector separated with a layer of silicone oil for reflecting the acoustic beam. (**b**) Piston UT is used with two reflective mirrors to reflect the laser beam. (**c**) Ring-shaped UT with an acoustic lens is used with a hole to transmit a laser beam. (**d**) Proposed concave transducer created using sol-gel composite spray technique. Pb(Zr_x_Ti_1−x_)O_3_ (0 ≤ x ≤ 1) (PZT)/PZT layer is fabricated on a curved substrate with a hole for transmitting a laser beam.

In OR-PAM, various designs of the OAT have been proposed to improve the efficiency of receiving PA signals. For example, prisms are used as a customized optical-acoustic combiner with an unfocused ultrasonic transducer, as shown in Figure 1a [1]. In this design, a silicone oil layer sandwiched by two prisms is utilized to achieve the confocal and coaxial alignment of optical and acoustic beams. The silicone layer is optically transparent but acoustically reflective. However, in this system, there is acoustic energy loss at the boundary between an acoustic lens, a silicone oil layer, and a medium. In addition, it is difficult to maintain a uniform thickness of the silicone oil layer because the thickness is easily influenced by the holder of prisms. Wang et al. have developed a different PA imaging system using a reflective objective and an ultrasonic transducer, as shown in Figure 1b [2]. The reflective objective has a long working distance and a large numerical aperture to achieve almost diffraction-limited optical focusing. A commercial transducer fabricated from a polyvinylidene fluoride (PVDF) polymer is placed underneath the objective to directly detect excited acoustic waves. This system can directly receive acoustic waves without obstacles. However, the sensitivity of PVDF is inferior to that of ceramic-type transducers such as Pb(Zr_x_Ti_1−x_)O_3_ (0 ≤ x ≤ 1) (PZT). Furthermore, an optical reflective mirror blocks the center part of an optical beam. This decreases the optical energy at the zero-order diffraction bright spot and degrades the resolution of PA images. In another design, a PZT transducer with a hole is used with an acoustic lens, as shown in Figure 1c [3]. This transducer can transmit a laser beam through the hole and receive a PA signal without a reflective objective. The acoustic lens is placed before the transducer to focus on PA signals. However, this system still experiences energy loss in the acoustic lens.

An OAT should satisfy the following requirements to improve the sensitivity and SNR of PA imaging: (1) the optical-acoustic arrangement should be aligned, (2) the shapes of an ultrasonic receiving surface and the wavefront of a PA signal should be the same to obtain PA signals in phase at each point on the receiving surface, and (3) the obstacles for optical and acoustic beams should be eliminated. Therefore, the objective of this study was to fabricate an optimized OAT for OR-PAM. The proposed OAT system is broadly illustrated in Figure 1d. This OAT can transmit lasers and directly receive acoustic waves.

Curved ultrasonic transducers with a hole at the center [8,9,10,11], which satisfy the above-mentioned requirements, have been mainly manufactured as high intensity focused ultrasound sensors [10,11]. However, they are difficult to manufacture and expensive. In this study, we fabricated an ultrasonic transducer suitable for PA imaging for the first time using a sol-gel composite material [12,13,14,15,16,17], which makes it easy to create a sensor with a curved shape, and evaluated its fundamental performance.

The sol-gel composite transducer is based on a sol-gel spraying technique, and it has been investigated in nondestructive testing applications [12,13]. The conventional sol-gel spraying technique uses only a sol-gel solution [18,19,20], whereas our method utilizes a sol-gel composite material, which is a mixture of a ferroelectric powder and a dielectric sol-gel solution. The piezoelectric layer fabricated by the sol-gel composite spraying method is composed of three phases: the ferroelectric powder phase, dielectric sol-gel phase, and air phase. The air phase is generated when the alcohol and water included in the sol-gel solution vaporizes during the firing process. This is described later. This method provides several advantages over conventional bulk PZT, PZT composites, and conventional sol-gel transducers in terms of the fabrication of the OAT. First, the concave piezoelectric layer can be fabricated by directly spraying the sol-gel composite material on the surface of a curved metal substrate. Second, backing and matching layers are not required because the PZT layer consists of tiny pores that decrease acoustic impedance. Third, the center frequency can be controlled by changing the number of spraying processes. Owing to these characteristics, this method is suitable for fabricating the ultrasonic transducer for PA imaging.

In a previous study, a flat surface sol-gel composite ultrasonic transducer was attached to a steel buffer rod. A probing end was machined into a hemispherical concave shape for the purpose of focusing, resulting in high spatial resolution with a lateral resolution of 0.19 mm and a focusing depth of 1.31 mm in the water at 23 °C [16]. However, curved surface sol-gel composite spraying transducers have never been fabricated for the purpose of focusing beams.

In another study, an ultrasonic sensor was fabricated on a stainless-steel plate with a hole in the center using the sol-gel spraying method, and laser irradiation and echo reception were performed simultaneously [14]. However, the distance from a focal point to each point of the sensor surface was inconsistent, resulting in long-tail echoes of approximately 5 μs. Next, another sensor with a curved surface was fabricated. Using the sensor and a preamplifier of 70 dB gain, an echo with a duration of approximately 0.5 μs and an amplitude of 4.7 V_p-p_ was obtained. However, the sensor size was 2.4 mm^2^, much smaller than that of the previous sensor, 300 mm^2^ [14]. Thereafter, the design was improved and a stronger echo was obtained [17]. This is described later. In this study, we developed an alternative method for fabricating a curved surface transducer using the sol-gel spraying method. We conducted experiments using a tissue phantom and performed three-dimensional maximum amplitude projection (MAP) to present the performance of the sensor.

The rest of this paper is organized as follows: Section 2 briefly describes the fabrication process of the transducer, its ultrasonic performance testing, and the experimental setup of PA imaging. The results are presented in Section 3. The discussion of the results is provided in Section 4. 

## 2. Materials and Methods 

### 2.1. Transducer Fabrication

**Figure 2:**
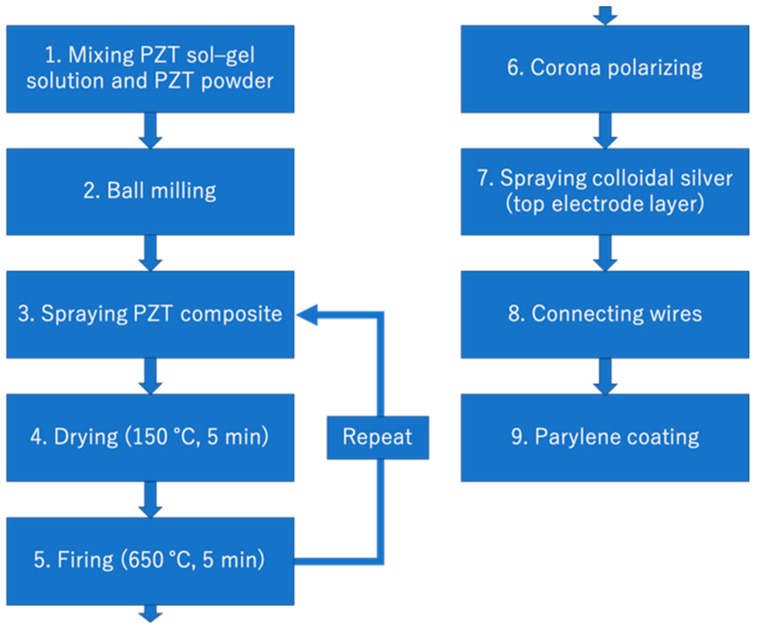
Flowchart of the fabrication process of a sol-gel composite PZT/PZT transducer.

We apply the sol-gel composite spraying technique to develop a concave-shaped single ultrasonic transducer. The general fabrication process of the sol-gel composite ultrasonic transducer is described below. First, a commercial PZT powder (Hizirco PZT L, Hayashi Chemical Industry Co., Ltd., Kyoto, Japan) [21] and a PZT sol-gel solution created in our laboratory are mixed at 1:2 ratio by weight using a ball milling machine for at least 24 h. Subsequently, the composite material is sprayed on a thin stainless-steel plate using a customized automatic coating machine at 25 °C. The thin stainless-steel plate is also used as a bottom electrode. After spraying, drying is performed at 150 °C for 10 min and firing is performed at 650 °C for 5 min. The spraying, drying, and firing processes are repeated until the PZT/PZT layer reaches a desirable thickness. After the PZT/PZT layer fabrication process, polarization is performed for 5 min at room temperature using a corona discharge device. After polarization, the piezoelectric d constant, d_33_, is measured using a d33 m (ZJ-3B, IACAS) to evaluate the fundamental performance of the transducer. The flowchart of the fabrication process is shown in Figure 2.

**Figure 3:**
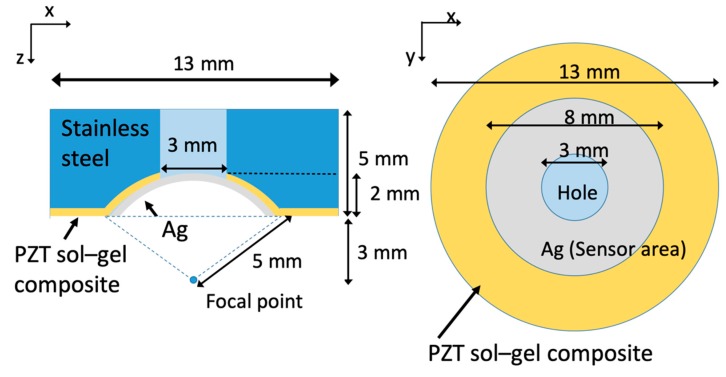
Design of fabricated transducer.

**Figure 4:**
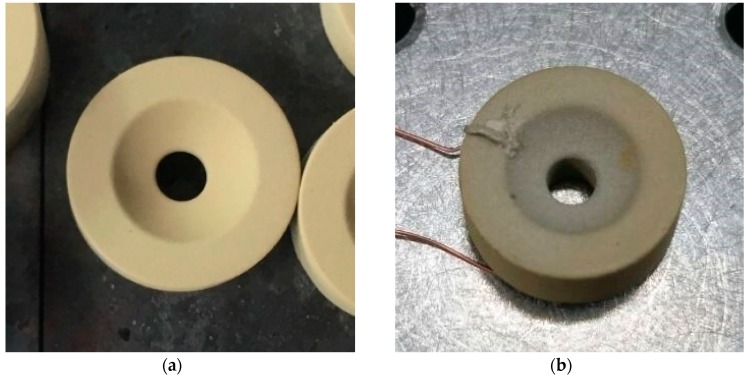
Fabricated transducer (**a**) after spraying, drying, and firing processes and (**b**) after connecting wires.

The sol-gel composite ultrasonic transducer is fabricated as follows to achieve the coaxial and confocal ability: Figure 3 shows the entire design of the transducer. The diameter of the rod is 13 mm, the height is 5 mm, and the rod consists of a hole with a diameter of 3 mm for transmitting a laser beam and scanning a small area. The concave surface with a curvature radius of 5 mm, which is sandwiched between colloidal silver and the stainless-steel rod, is the active transducer area. The distance between each point on the surface and the focal point is 5 mm, and the depth from the bottom of the transducer to the focal point is 3 mm. The optical and acoustic focal points are set at the same position to achieve coaxial and confocal alignment. Figure 4a shows the piezoelectric layer of the transducer. The piezoelectric constant, d_33_, is approximately 76 pC/N. The top electrode layer is fabricated by spraying colloidal silver on the piezoelectric layer using an airbrush. After spraying colloidal silver, the substrate is dried at 150 °C for 1 min using a heat gun. Next, two wires are bonded on the bottom and top electrodes by utilizing an electrically conductive adhesive. The stainless-steel rod is used as a substrate. Consequently, the transducer is coated with parylene for waterproofing [22]. Figure 4b shows the finished transducer.

### 2.2. Pulse-Echo Experimental Design

**Figure 5:**
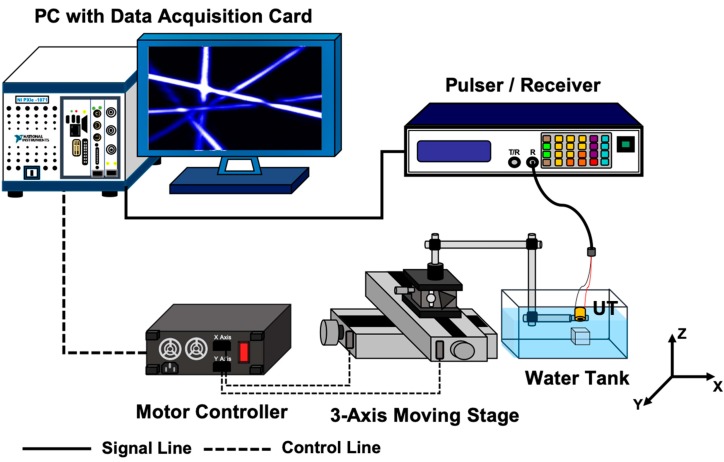
Setup of the pulse-echo experiment using fabricated transducer. The fabricated transducer is moved by the three-dimensional stage and ultrasonic pulses are excited at each point. The moving pitch is approximately 13.3 μm and 500 μm in the lateral and axial directions, respectively.

The diagram of a pulse-echo experimental setup is shown in Figure 5. In the experiment, the fabricated transducer was fixed by a clamp with a 3-axis stage and moved to perform scans in the lateral and axial directions. A pulser/receiver (MODEL 5800, Olympus Corp., Tokyo, Japan) was used for transmitting and receiving ultrasound. The stage and pulser/receiver were controlled using LABVIEW (National Instruments, Austin, TX, USA). Ensemble average processing was applied to the obtained echo signals, with 100 times at each spatial point. In receiving, preamp gain was set as 40 dB, high-pass filter frequency was set as 1 MHz, and low-pass filter frequency was set as 10 MHz.

## 3. Results

### 3.1. Fundamental Performance of Fabricated Transducer

**Figure 6:**
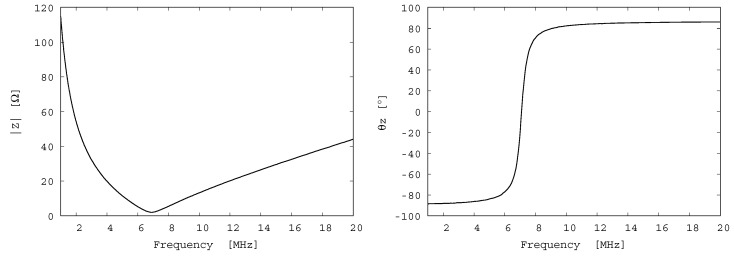
Electrical impedance of the fabricated transducer. Resonant frequency is approximately 7.0 MHz.

To evaluate the performance of the fabricated transducer, its electrical impedance was measured using a network analyzer (E5061B, Keysight Technologies, Inc., Santa Rosa, CA, USA). The measured frequency characteristic of the electrical impedance is shown in Figure 6. According to this figure, the resonant frequency is 7 MHz. However, an antiresonant frequency is not seen below 30 MHz. Therefore, it can be predicted that the antiresonant frequency is considerably higher than 30 MHz. 

**Figure 7:**
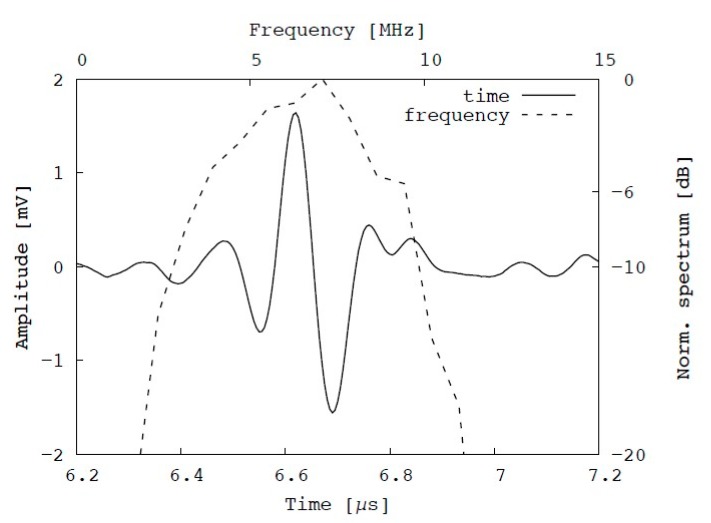
Pulse-echo signal obtained from copper wire using fabricated focused transducer through pulse-echo testing in time (solid line) and frequency (dashed line) domains.

**Figure 8:**
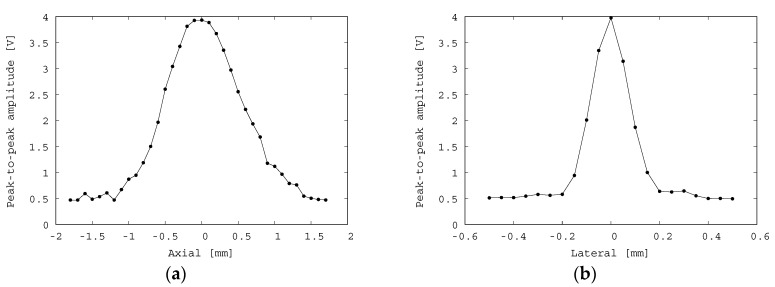
(**a**) Axial and (**b**) lateral intensity profiles of the fabricated focused transducer in the pulse-echo experiment. The full width at half maximum (FWHM) is approximately 1.25 mm and 0.2 mm in the axial and lateral directions, respectively.

A pulse-echo experiment was conducted with the experimental setup as described in Figure 5 to characterize the acoustic resolution performance of the fabricated transducer. In the experiment, the fabricated transducer was fixed by a clamp with a 3-axis stage and moved to perform scans in the lateral and axial directions. A transparent phantom was constructed, which consisted of a copper wire with a diameter of 0.15 mm embedded in transparent agar with a sound velocity of approximately 1500 m/s. The agar phantom was placed in a water tank, and the echo signals from the copper wire were collected. An example of an echo signal from the copper wire is shown in Figure 7. The center frequency is approximately 7 MHz, and its −6 dB frequency bandwidth is 6 MHz (3.5–9.5 MHz). Hence, the relative frequency bandwidth is 86%. This bandwidth is fairly broad considering that matching and backing layers were omitted in the fabricated transducer. Figure 8 illustrates the axial and lateral resolution obtained by measuring the amplitudes of the echoes from the copper wire by moving the 3-axis stage. The maximum peak-to-peak amplitude is approximately 4 V_p-p_, and the full width at half maximum (FWHM) is approximately 1.25 mm and 0.2 mm in the axial and lateral directions, respectively. It is found that a satisfactory large amplitude and small focal size are obtained owing to the curved shape of the fabricated transducer. 

### 3.2. Photoacoustic Visualization

**Figure 9:**
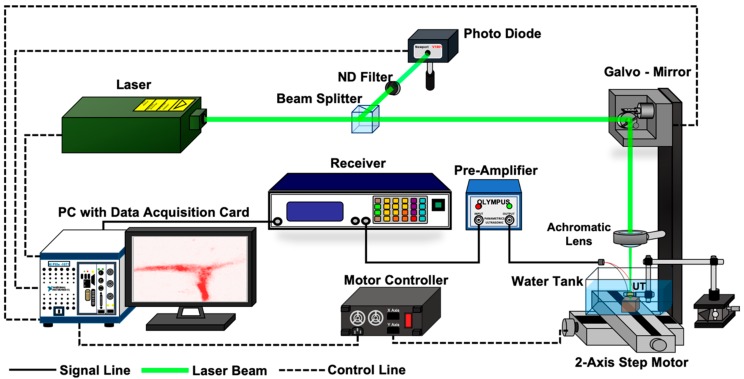
Experimental setup for photoacoustic (PA) imaging using fabricated focused transducer and conventional optic-acoustic transmitter (OAT).

**Figure 10:**
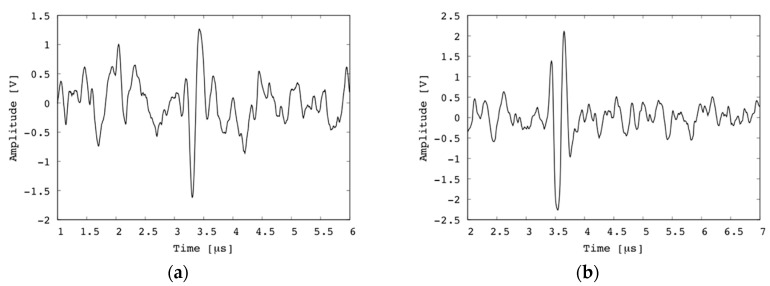

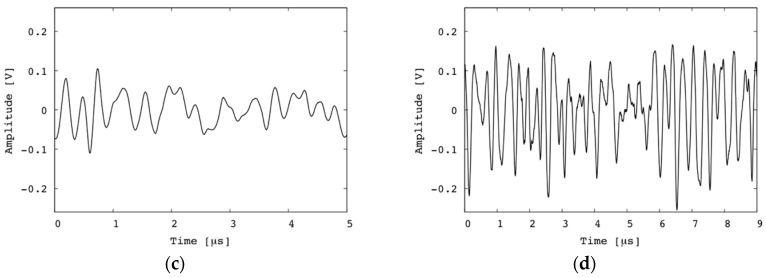
PA signals obtained from (**a**) copper wire and (**b**) hair using fabricated focused transducer, (**c**) copper wire and (**d**) hair using conventional OAT.

The experimental setup for PA imaging is shown in Figure 9. In this experiment, a pulsed fiber laser (GLPM-10, IPG Photonics Corp., Massachusetts, Japan) with a wavelength of 532 nm and an input beam diameter of 5 mm was used. The focal length of the objective lens was 30 mm. The theoretical spot size of the laser was 5.5 μm. The pulse energy out of the objective lens was set as 0.6 μJ. This energy is significantly lower compared to previous studies. This is described in detail later. The pulse repetition frequency (PRF) of the emitted pulse was set as 50 kHz. A pulsed laser was generated, and it passed through a beam splitter, which was used for obtaining a trigger signal. Subsequently, the laser was reflected at a mirror, propagated through an achromatic lens (AC254-030-A, Thorlabs, Inc., Newton, NJ, USA), and finally focused at a point in a specimen. The emitted PA signals were received by the fabricated transducer. A conventional OAT consisting of an acoustic lens with a focal length of 12.7 mm, reflecting prisms, and an unfocused transducer (V384, Olympus Inc.) with a center frequency of 3.5 MHz was used as a reference, as shown in Figure 1a. The obtained signals were amplified by a pulser/receiver (MODEL 5800, Olympus Inc.) and a preamp (MODEL 5678, Olympus Inc.). The total gain was 60 dB, the cutoff frequency of the high-pass filter was 1 MHz, and the cutoff frequency of the low-pass filter was 10 MHz. The obtained signals were digitized at a sampling rate of 400 MHz and transferred to a computer, where an image was formed. In addition, ensemble average processing was applied to 20 signals to obtain smooth signals. As a specimen, a copper wire with a diameter of 0.15 mm and a human hair with a diameter of approximately 50 μm were separately embedded in transparent agar and fixed in the water tank. The signals obtained using the fabricated transducer and conventional OAT are shown in Figure 10. As shown in Figure 10a,b, the amplitude of the PA signal obtained using the fabricated focused transducer is 2.8 V_p-p_ for the copper wire and 4.4 V_p-p_ for the human hair. On the other hand, no peak is observed in the waveform obtained using the conventional OAT, as shown in Figure 10c,d.

Although the waveform obtained by the fabricated focused transducer has a larger amplitude of noise signal than that of the conventional OAT, the ratio of the PA signal to the noise is 1.6 for copper wire and 3.4 for human hair. On the other hand, the SNR of the conventional OAT must be below unity because the conventional OAT could not obtain a PA signal.

**Figure 11:**
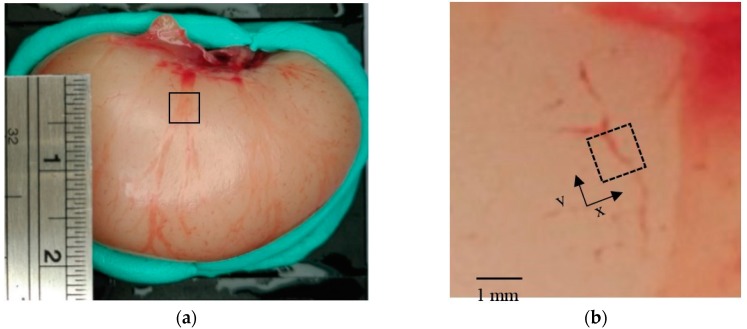
(**a**) Photographs of chicken testicle and (**b**) magnified view of the region bounded by the solid black line in (**a**). Black-dashed box in (**b**) indicates a scan area with a size of 1 × 1 mm^2^ (pixel size: 100 × 100). Vessel diameter is less than 75 μm.

**Figure 12:**
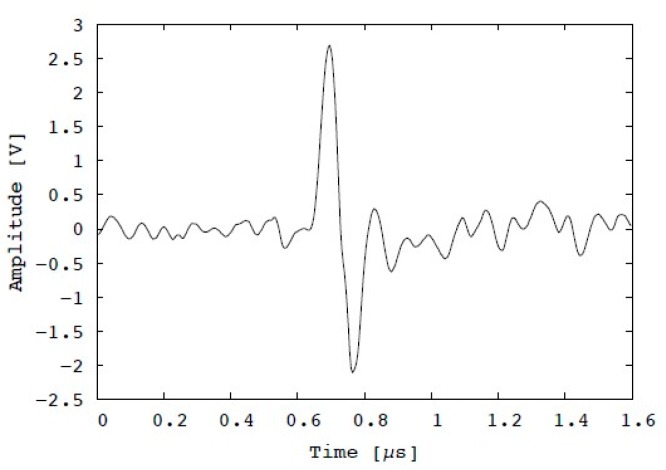
Example of PA signal obtained from chicken testicle.

A chicken testicle was used as a specimen to evaluate the transducer for biomaterial imaging, as shown in Figure 11. The scanning area was set as 1 × 1 mm^2^ (the number of pixels was 100 × 100). The blood vessels on the surface with a diameter of less than 75 μm were visualized. Figure 12 illustrates an example of the PA signal obtained from the blood vessels. The peak-to-peak amplitude is approximately 4.5 V, and the SNR is 13 dB. 

**Figure 13:**
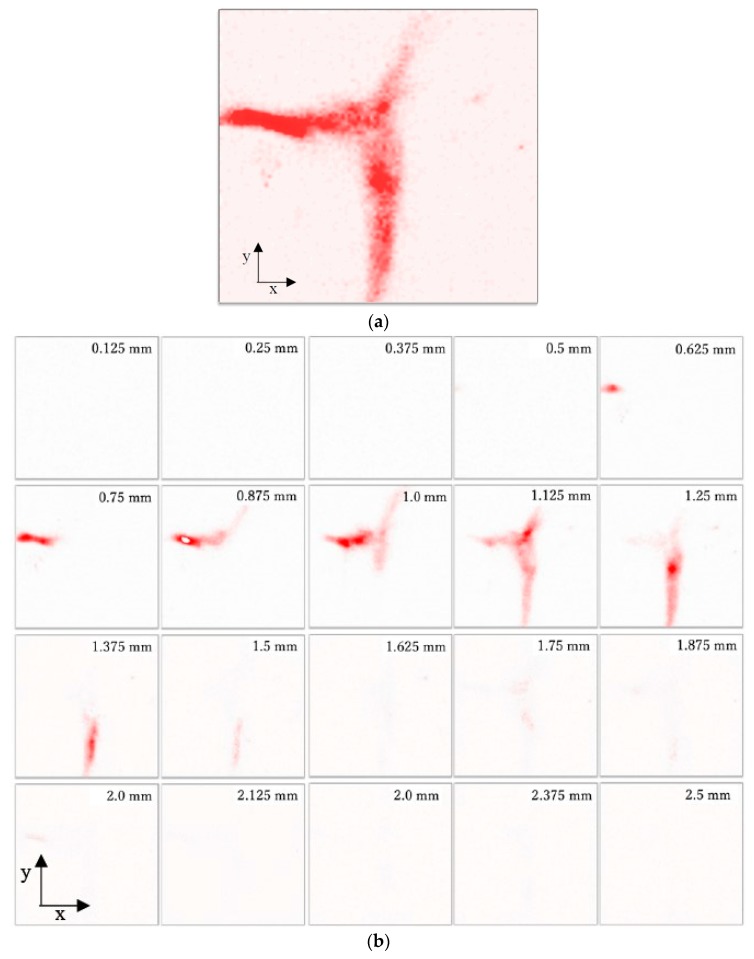

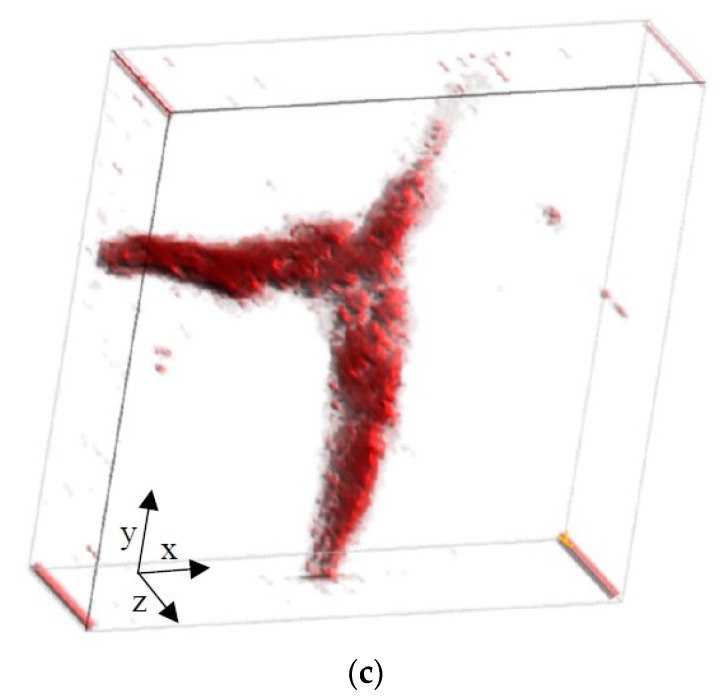
Reconstructed maximum amplitude projection (MAP) images of chicken testicle. (**a**) Two-dimensional MAP image, (**b**) two-dimensional MAP at each depth, and (**c**) three-dimensional MAP image.

In the imaging process, MAP was applied to the obtained PA signals. The maximum amplitude (*z*-coordinate) of a PA signal at each position scanned in *x*-*y* coordinates was calculated as

(1) $$\mathrm{MAP}\left(x,y\right)\;=\;\underset{0\leq i\leq N}{\max}s\left(x,y,i\right),$$

where *s* is the PA signal obtained from the specimen, *x* and *y* are the coordinates, *i* is discrete time, and *N* is discrete measured time. First, one maximum value at each pixel was selected for the entire measurement time range to create a two-dimensional MAP image, which is illustrated in Figure 13. The MAP image captures the branching of a blood vessel, which is similar to the shape in the optical image shown in Figure 11. Subsequently, to visualize the specimen three-dimensionally, the obtained PA signal was divided at intervals of 0.125 mm and 20 planes were obtained. Each maximum value was stacked and visualized three-dimensionally. The two-dimensional MAP image obtained at each depth and the three-dimensional MAP image obtained by stacking these images are shown in Figure 13a,b, respectively. Even though the conventional OAT with the transducer (V384, Olympus Inc.) was used for measurement as a reference, the PA signal could not be obtained owing to significantly low signal energy that was below the noise level.

## 4. Discussion and Conclusions

In this study, a sol-gel composite ultrasonic transducer was successfully fabricated on a curved-surface stainless-steel substrate to produce a newly designed OAT. The substrate was machined into a concave shape to focus at a point 5 mm from each surface point, and a 3-mm-diameter hole was drilled in the center of the substrate to allow the focused laser beam to pass through. 

Using the fabricated sol-gel composite ultrasonic transducer in the pulse-echo mode, the duration of the echo obtained was approximately 0.2 μs, significantly shorter than the 5 μs duration of the previously fabricated flat-type transducer. The FWHM was 1.25 mm in the lateral direction and 0.2 mm in the axial direction. This is much larger than the laser focal zone. In particular, the transducer can visualize in the axial range of 1.25 mm without moving the laser focus in the depth direction.

PA signals were obtained by using the fabricated sol-gel composite ultrasonic transducer. Since the lateral FWHM of the transducer (0.2 mm) was larger than the spot size of the laser (5.25 μm), the laser beam can tilt in the range of the lateral FWHM of the transducer using the Galvano-mirror. However, to obtain PA images with a diameter larger than 0.2 mm, the OAT was fixed with the stage to align the ultrasonic and laser beams on the same axis, and the phantom was moved by a 2-axis step motor. As a result, we succeeded in visualizing a blood vessel with a diameter of 75 μm within a collected image range of 1 × 1 mm^2^. The branching of the blood vessel can be observed in different depth and stacked as a 3D image.

On the other hand, using the conventional OAT in which combined the acoustic lens, reflecting prisms, and the commercial ultrasonic transducer, the PA signal for the same blood vessel was buried in noise, and could not be acquired. In addition, it may hurt the specimen when transmit more energy to obtain the stronger PA signal.

The advantages of this system are as follows: the axes of the laser beam and ultrasonic beam can be aligned with the focus point without using reflective devices such as prisms and mirrors, the fabricated transducer is curved so that the PA signals can be collected with the same phase from the focal point so an acoustic lens is not necessary, and the transducer can be placed closer to the focal point so that the ultrasonic propagating attenuation can be suppressed. From these aspects, the sol-gel composite ultrasonic transducer has potential as a new alternative for PA imaging to PVDF and other piezoelectric transducers.

With respect to ease of fabrication, only the machining processing of the substrate is unique to the fabrication process of the transducer presented in this paper. The other processes are the same as for other sol-gel composite ultrasonic transducers. This means that many different types and shapes of sol-gel composite ultrasonic transducers can be sprayed and manufactured together, thereby reducing production costs.

However, the technique has drawbacks in real-time visualization. Three-dimensional MAP images are obtained by calculating the MAP at regular intervals in the depth direction. If the horizontal resolution of the proposed system is significantly smaller than the region of interest, the number of scans increases considerably, and the process becomes time-consuming. This problem must be resolved to perform real-time visualization using the proposed transducer. 

Widening the acquisition area of the sol-gel composite ultrasonic transducer is an alternative method of achieving real-time visualization. The curvature of the transducer surface can be optimized to extend the focal zone of the transducer, and hence, the acquisition area can be widened. However, this decreases the amplitude of the PA signals obtained from each point. To compensate for this, the sensitivity of the sol-gel composite ultrasonic transducer should be further improved to increase the acquisition area of PA signals. If the acquisition area is wide, scanning a laser beam only within the acquisition area at high speed will lead to real-time visualization. Annular and two-dimensional arrays are good alternatives for efficiently obtaining PA signals. An array can be created for our transducer by patterning the top electrodes. However, the fine patterning of the top electrodes and extensive wiring are required for performing real-time PA imaging. In the future, the fabrication of various shapes of the array transducer will be investigated.
